# Targeted Drug-Resistance Testing Strategy for Multidrug-Resistant Tuberculosis Detection, Lima, Peru, 2005–2008

**DOI:** 10.3201/eid1703.101553

**Published:** 2011-03

**Authors:** Gustavo E. Velásquez, Martin Yagui, J. Peter Cegielski, Luis Asencios, Jaime Bayona, Cesar Bonilla, Hector O. Jave, Gloria Yale, Carmen Suárez, Sidney Atwood, Carmen C. Contreras, Sonya S. Shin

**Affiliations:** Author affiliations: Brigham and Women’s Hospital, Boston, Massachusetts, USA (G.E. Velásquez, S. Atwood, S.S. Shin);; Instituto Nacional de Salud, Lima, Peru (M. Yagui, L. Asencios);; Centers for Disease Control and Prevention, Atlanta, Georgia, USA (J.P. Cegielski);; Socios En Salud, Lima (J. Bayona, C.C. Conteras, S.S. Shin);; Partners In Health, Boston (S.S. Shin);; Dartmouth College, Hanover, New Hampshire, USA (J. Bayona);; Ministerio de Salud del Perú, Lima (C. Bonilla, H.O. Jave);; Dirección de Salud V Lima Ciudad, Lima (G. Yale);; Dirección de Salud IV Lima Este, Lima (C. Suárez)

**Keywords:** Bacteria, tuberculosis and other mycobacteria, MDR TB, resistance, multidrug resistant tuberculosis, testing strategy, Peru, research

## Abstract

The Peruvian National Tuberculosis Control Program issued guidelines in 2006 specifying criteria for culture and drug-susceptibility testing (DST), including district-level rapid DST. All patients referred for culture and DST in 2 districts of Lima, Peru, during January 2005–November 2008 were monitored prospectively. Of 1,846 patients, 1,241 (67.2%) had complete DST results for isoniazid and rifampin; 419 (33.8%) patients had multidrug-resistant (MDR) TB at the time of referral. Among patients with new smear-positive TB, household contact and suspected category I failure were associated with MDR TB, compared with concurrent regional surveillance data. Among previously treated patients with smear-positive TB, adult household contact, suspected category II failure, early relapse after category I, and multiple previous TB treatments were associated with MDR TB, compared with concurrent regional surveillance data. The proportion of MDR TB detected by using guidelines was higher than that detected by a concurrent national drug-resistance survey, indicating that the strategy effectively identified patients for DST.

## Abstract 2

Running head: Targeted Drug-Resistance Testing Strategy for MDR TB

Multidrug-resistant tuberculosis (MDR TB) is defined as infection with *Mycobacterium tuberculosis* with in vitro resistance to at least isoniazid and rifampin. The incidence of MDR TB disease was estimated to be 0.5 million in 2007, with a prevalence of as many as 2 million cases worldwide (*1*). Although no single best approach to MDR TB treatment has been recognized, rapid drug-susceptibility testing (DST) and prompt initiation of effective treatment are achievable goals. Ideally, treatment is based on timely, accurate DST, but if universal DST is not possible or not yet available, the national TB control program can prioritize patients at increased risk for MDR TB.

Rapid DST methods should minimize delays to initiation of appropriate treatment (*2**,**3*). Numerous assays have been developed that have characteristics suitable for use in low-income settings, including low cost, modest technical demand, and high accuracy (*4**–**6*). The nitrate reductase assay (NRA), also known as the Griess method, has demonstrated acceptable sensitivity, specificity, and speed compared with conventional DST and rapid phenotypic DST methods (*7**,**8*). This phenotypic assay was developed in Russia as a low-cost drug-susceptibility test that can be used in areas of moderate technical capacity (*9*). The method is based on a nitrate-reductase colorimetric reaction that uses Lowenstein-Jensen (LJ) medium prepared with antimicrobial drugs (*9*). Although initially validated as an indirect method, it was implemented as a direct method by the Peruvian National Institute of Health (INS) (*10*). The NRA yields drug-susceptibility information to isoniazid and rifampin 21–28 days after inoculating a smear-positive sputum sample (direct method) (*10*) or 8–10 days after obtaining a positive culture (indirect method). The pooled sensitivity and specificity of the NRA (on culture isolates and sputum) have been reported to be 97% and 100% for rifampin and 96% and 99% for isoniazid (*11*). The pooled sensitivity and specificity of direct NRA have been reported to be 99% and 100% for rifampin and 94% and 100% for isoniazid (*12*). A recent comparison of 4 rapid DST methods with conventional DST in the context of a clinical trial suggested they may be cost-effective when compared with other health interventions (*13*). On the basis in part of these data, the World Health Organization (WHO) recently endorsed the use of NRA for screening patients at risk of MDR TB (*14*).

Despite the development of promising commercial and noncommercial rapid methods for MDR TB diagnosis, how to implement those methods under program conditions remains largely unaddressed. DST performance in validation studies differs greatly from performance integrated within a TB control program. Furthermore, the performance of any method under program conditions depends not only on assay characteristics, but also on the assets of the laboratory network and National Tuberculosis Control Program (NTP) guidelines which define criteria for performing DST.

To address this gap, we evaluated the effects of a programmatic strategy for rapid screening for MDR TB among risk groups specified by the Peruvian NTP in April 2006 (*15*). At that same time, decentralized, district-level MDR TB screening was pilot tested in 2 district laboratories in Lima, Peru. In collaboration with the Peruvian NTP and the Peruvian National Reference Laboratory (NRL), we evaluated the effectiveness of these combined strategies for detecting MDR TB. We report the proportion of drug resistance among risk groups based on screening high-risk patients as defined by explicit criteria, including rapid methods of DST in one of the first countries to implement this strategy.

## Materials and Methods

### Study Setting and Program Description

The prevalence of TB in Peru was 38,000 cases, and the incidence of TB in Peru was 126 cases/100,000 population per year, by 2007 estimates (*16*). The most recent national surveillance data indicate 5.3% of new TB cases and 24% of previously treated TB cases are MDR TB (*16*). In 1996, a collaborative effort to provide individualized treatment by using second-line drugs for MDR TB in northern Lima was established by Partners In Health, Socios En Salud, Harvard University, the Massachusetts State Laboratory Institute, the Peruvian NTP, and the Peruvian INS (*4*). In October 1997, the Peruvian NTP began a standardized treatment regimen including directly observed therapy with second-line drugs for patients in whom first-line drugs failed (*17*). Only 48% of these patients were treated successfully. During 2005 and 2006, the Peruvian INS and NTP transferred the capacity for DST to first-line TB drugs from the central level at the NRL to 2 district-level reference laboratories in Lima as a prelude to decentralizing these services to all major provinces (*4*). Simultaneously, the NTP issued national guidelines codifying criteria for MDR TB screening on the basis of known and suspected risk factors for MDR TB. In addition, these guidelines recommended the use of a more aggressive empiric MDR TB treatment regimen, including 5 second-line drugs for those persons with suspected MDR TB pending DST results. These programmatic and laboratory efforts resulted in an integrated strategy to diagnose and treat MDR TB cases in a timely and aggressive manner. During this period, we evaluated the effectiveness of selection criteria for DST in the first 2 laboratories (for health districts Lima Ciudad and Lima Este) where the NRA was implemented.

For persons in whom DST to first-line drugs confirmed drug resistance to isoniazid or rifampin, or both, the same isolate would be sent to the NRL for testing to a full panel of 5 first-line drugs and 5 second-line drugs. DST results were conveyed to health center providers by paper or electronic communication, and patients were evaluated with DST results to determine whether further regimen modification was needed (*18*). Details of treatment regimens have been described elsewhere (*19*). All patients diagnosed with TB were provided directly observed therapy free of charge through the NTP.

### Study Patients and Enrollment Period

The patients enrolled in this cohort had suspected TB with respiratory symptoms living in 2 districts of Lima, Peru, Lima Ciudad or Lima Este, who met Peruvian NTP guidelines for DST referral as elaborated in Table 1. There were no exclusion criteria.

**Table 1:** Criteria for drug-susceptibility testing referral per Peruvian National Tuberculosis Control Program guidelines*

A. Newly diagnosed smear- or culture-positive patients at risk for MDR TB. Persons were eligible for enrollment if they were 1) diagnosed with smear-positive pulmonary TB, 2) had no history of TB, and 3) had >1 of the following risk factors:
1. Household contact of patient with documented MDR TB
2. Household contact of patient in treatment with second-line drugs
3. Household contact of patient who showed failure of TB therapy
4. Household contact of patient who died of TB within the past 2 years
5. HIV-positive by ELISA and Western blot confirmation
6. Diabetes mellitus
7. Health care worker, regardless of health care field, in the past 2 years
8. Student of health sciences in the past 2 years
9. Employee of the penitentiary system
10. Chronic treatment with corticosteroids
11. Other condition of immunosuppression
12. Adverse reaction to TB medications requiring a change in regimen
13. Hospitalization for any indication in the past 2 years lasting >15 days
B. Patients in whom first-line or second-line therapy may be failing. Persons were eligible for enrollment if they were 1) currently receiving first-line or second-line treatment, and 2) had a sputum sample collected after >2 months of treatment that was smear positive (i.e., monthly sputum collected between months 2 and 6)
C. Patients who had received >1 previous treatment and who did not have documented MDR TB. This included persons who:
1. Abandoned any previous regimen and now presented for retreatment
2. Relapsed after completion of any previous regimen within 6 months
3. Unsuccessful treatment with any previous regimen
4. Received multiple courses of TB treatment
5. Had a history of private or auto-administered treatment
D. Newly diagnosed smear-negative patients at risk for smear-negative MDR TB. Persons were eligible for enrollment if they were 1) suspected to have active pulmonary TB, 2) were smear negative, 3) had no history of TB therapy, and 4) had >1 of the following risk factors:
1. Pediatric household contact of patient with documented MDR TB
2. Pediatric household contact of patient who died of tuberculosis within the past 2 years
3. HIV positive by ELISA and Western blot confirmation

*MDR, multidrug resistant; TB, tuberculosis.

### Enrollment Methods

Patients were identified by health care workers at their local health care establishments, and their sputum samples were sent to the reference laboratory for DST. Because all sputum samples for DST were sent to the district laboratories, subjects eligible for enrollment were identified by this referral. Study personnel visited each district laboratory on a regular basis to review sample referrals and confirm that all eligible subjects had been identified. Large, busy health centers were visited weekly and smaller, rural health centers were visited at least monthly for review of patient medical records. This method was used to confirm that all patients were included who were eligible without duplications. In Lima Ciudad, patients were enrolled from January 2005 through March 2008. In Lima Este, patients were enrolled from May 2005 through May 2008.

### Drug Susceptibility Methods

The scale-up of MDR TB laboratory services in Peru, including expansion of the BACTEC-460 system (Becton, Dickinson and Company, Sparks, MD, USA) and NRA (Griess) method for rapid first-line DST are described elsewhere (*4**,**5**,**20*). The Peruvian NRL performed BACTEC-460 culture and DST on paucibacillary and smear-negative samples, prioritizing pediatric cases, HIV-positive persons, and health care workers. The scale-up of second-line conventional DST at the NRL by the indirect agar plate proportion method has also been described elsewhere (*4*). The district reference laboratories cultured sputum specimens processed with 4% NaOH on Ogawa medium without centrifugation. During 2005, BioSafety Level 3 working conditions were established in 2 district reference laboratories, and these laboratories implemented DST on LJ medium for first-line drugs by using the indirect proportion method. Each procedure was validated by each laboratory through comparison with the Peruvian NRL and the Massachusetts State Laboratory Institute. Subsequently, the NRA was implemented in Lima Ciudad in December 2005 and in Lima Este in March 2007. The study spanned pre- and postintervention periods: January 2005 through November 2008 for Lima Ciudad and May 2005 through November 2008 for Lima Este. The NRA was used for rapid screening of smear-positive specimens from patients with the risk factors outlined in Table 1. Sputum specimens were processed with 2% NaOH/n-acetyl-l-cysteine, centrifuged at 3,000 × *g*, cultured on LJ medium, and simultaneously inoculated on modified LJ medium for the NRA to detect isoniazid and rifampin resistance.

### Data Collection

A team of trained data recorders prospectively collected data using standardized forms. Sources of data included patient charts and laboratory registries and databases. HIV status was routinely recorded for TB patients. Available chest radiographs were reviewed by TB physicians who used standardized criteria to identify the type and location of radiographic abnormalities. Chest radiograph data were included if the radiograph was performed <1 year before the enrollment date or <1 month after the enrollment date. At baseline, clinical and sociodemographic data were recorded in addition to the risk factors outlined in Table 1. Data were entered into an Epi Info version 3.4.3 database (Centers for Disease Control and Prevention [CDC], Atlanta, GA, USA).

In addition to the data prospectively collected in this study, we used national and regional data from Peruvian NTP surveillance on drug resistance that were collected as part of the WHO Fourth Global Report on Anti-Tuberculosis Drug Resistance in the World (*21*). Surveillance and laboratory methods are described in more detail in the WHO report (*21*). For national data, we used all data in the NTP surveillance; for regional data, we included samples collected from health establishments served by the Lima Ciudad and Lima Este laboratories, which corresponded to the same catchment area as our study (Ministerio de Salud, unpub. data).

Patients were identified as having MDR TB if they had a positive culture for *M. tuberculosis* and DST results showed resistance to at least isoniazid and rifampin. Extensively drug-resistant TB was defined as resistance to at least isoniazid, rifampin, any fluoroquinolone, and >1 of 3 injectable second-line drugs (amikacin, capreomycin, or kanamycin). Monoresistance was defined as drug-resistance to isoniazid or rifampin, but not both drugs. Patients were considered to have drug-susceptible TB if their isolate was susceptible to both isoniazid and rifampin. Baseline refers to DST data at the time of referral, i.e., testing performed on sputum samples collected within 30 days of study enrollment. If baseline drug resistance data were not available for both isoniazid and rifampin (e.g., because of a culture-negative sample or because of contamination), the patient was considered to have no DST result.

### Statistical Analysis

All analyses were performed by using STATA/IC version 10.1 (StataCorp LP, College Station, TX, USA). The χ^2^ test or Fisher exact test was used to calculate p values, when appropriate. Point and interval estimation for the odds ratio were performed by using the Woolf procedure or the exact method, when appropriate. The Breslow-Day test for homogeneity was used to explore for effect modification. All statistical tests were 2-sided, and significance was set at α = 0.05.

### Ethical Approval

The prospective observational cohort study providing data for this analysis was approved by institutional review boards at Brigham and Women’s Hospital and the Peruvian INS. An institutional review board amendment describing the aims of this analysis was approved by the Peruvian INS on October 15, 2008, and by the Partners Human Research Committee for Brigham and Women’s Hospital on September 23, 2008. This activity was approved by CDC as program evaluation and not as human subject research.

## Results

A total of 1,846 patients were enrolled during the study period. Among these, 605 (32.8%) did not have baseline DST results, either due to a nonviable sample (99.2%) or incomplete resistance data for both isoniazid and rifampin (0.8%). The remaining 1,241 (67.2%) patients constitute the cohort for analysis presented here.

Of these 1,241, 419 (33.8%) had baseline MDR TB, among whom 195 (46.5%) had never been treated for TB and 224 (53.5%) had a history of previous TB treatment. Eight patients had extensively drug-resistant TB; 1 was a medical student, 1 had received prior self-administered treatment, 2 had household contacts (1 was a pediatric patient and the other was an adult), and 4 were identified as suspected category I failures, i.e., failure of first-line treatment for new patients. Of these 8 case-patients, only 1 (who had received self-administered treatment) had completed previous treatment.

Descriptive characteristics of the cohort are shown in Table 2. Compared with patients with drug-susceptible TB, those with MDR TB were younger, more likely to be single, more educated, and less likely to have ever smoked. Clinically, they were less likely to have been tested by using the BACTEC-460 system and more likely to have hemoptysis. MDR TB patients and patients with drug-susceptible TB did not significantly differ with respect to their year of enrollment, gender, and history of TB treatment. Compared with patients with drug-susceptible TB, patients with monoresistant TB were younger and more likely to be single.

**Table 2:** Demographic and clinical characteristics of patients with tuberculosis, by drug-resistance status, Lima, Peru, 2005–2008*

Variable	Susceptible to INH and RIF, n = 661	Monoresistant to INH or RIF, n = 161	MDR TB, n = 419	Total, N = 1,241
DST method				
Griess	318 (48.1)	87 (54.0)	208 (49.6)	613 (49.4)
Conventional	270 (40.9)	56 (34.8)	185 (44.2)	511 (41.2)
BACTEC	63 (9.5)	17 (10.6)	**23 (5.5)†**	103 (8.3)
Griess/BACTEC	10 (1.5)	1 (0.6)	3 (0.7)	14 (1.1)
Year of enrollment				
2005	175 (26.5)	44 (27.3)	130 (31.0)	349 (28.1)
2006	233 (35.3)	47 (29.2)	127 (30.3)	407 (32.8)
2007	178 (26.9)	48 (29.8)	106 (25.3)	332 (26.8)
2008	75 (11.4)	22 (13.7)	56 (13.4)	153 (12.3)
Age, y, mean ± SD	35.6 ± 15.2	**32.8 ± 15.1†**	**29.7 ± 13.1‡**	33.2 ± 14.8
Female sex	236 (35.7)	54 (33.5)	144 (34.4)	434 (35.0)
Married or lived together	272 (41.2)	**48 (29.8)§**	**136 (32.5)§**	456 (36.7)
Unemployed, n = 1,239	257 (38.9)	62 (38.8)	155 (37.0)	474 (38.3)
Did not begin secondary level education, n = 1,235	150 (22.9)	31 (19.3)	**67 (16.0)§**	248 (20.1)
Tobacco use (ever), n = 1,240	191 (28.9)	48 (29.8)	**97 (23.2)†**	336 (27.1)
Alcohol use or abuse (ever), n = 1,240	257 (38.9)	61 (37.9)	148 (35.3)	466 (37.6)
Illicit drug use (ever)	131 (19.8)	38 (23.6)	77 (18.4)	246 (19.8)
Weight loss, n = 1,237	543 (82.5)	126 (78.8)	330 (78.8)	999 (80.8)
Dyspnea, n = 1,238	118 (17.9)	21 (13.1)	81 (19.3)	220 (17.8)
Hemoptysis, n = 1,239	28 (4.3)	9 (5.6)	**38 (9.1)§**	75 (6.1)
Cavitary lesion on chest radiography, n = 1,207	199 (16.5)	43 (3.6)	144 (11.9)	386 (32.0)
Low BMI, n = 1,233	203 (30.8)	55 (34.8)	133 (32.1)	391 (31.7)
Previous TB treatment	328 (49.6)	77 (47.8)	224 (53.5)	629 (50.7)
Type of TB				
Pulmonary only	650 (98.3)	155 (96.3)	414 (98.8)	1219 (98.2)
Extrapulmonary	11 (1.7)	6 (3.7)	5 (1.2)	22 (1.8)

*Values are no. (%) except as indicated. **Boldface** indicates significant difference in statistical comparison of baseline characteristics in the corresponding drug-resistance group to drug-susceptible cases. INH, isoniazid; RIF, rifampin; MDR TB, multidrug-resistant tuberculosis; DST, drug-susceptibility testing; BMI, body mass index. †p<0.05. ‡p<0.001. §p<0.01.

The most frequent risk factors prompting referral for DST among patients with new smear-positive TB were being adults with a household contact with known or suspected MDR TB (32.1%), diabetes mellitus (20.0%), and suspected category I failures (19.5%). Among previously treated patients with smear-positive TB, those with multiple (>2) treatments (43.2%), adult household contact (18.6%), default of category I treatment (16.8%), and previously self-administered treatment (14.9%) were most frequently referred for DST. Among all patients with smear-positive TB, a single risk factor was identified in 485 (43.54%) patients, whereas 382 (34.29%), 205 (18.40%), 38 (3.41%), and 4 (0.36%) had 2, 3, 4, and 5 risk factors, respectively (data not shown).

The prevalence of MDR TB in Peru in 2007 among all TB patients, previously treated TB patients, and new TB patients is shown in Table 3 (*21*). In this national surveillance report, 8.3% of all TB patients, 5.2% of new TB patients, and 24.2% of previously treated TB patients in Peru were estimated to have MDR TB (*21*). Limiting surveillance data to the 2 districts where our cohort was enrolled, 12.4% of all TB patients, 9.9% of new TB patients, and 24.0% of previously treated TB patients had MDR TB (Ministerio de Salud, unpub. data). In our cohort of 1,241 subjects, 33.8% of all patients, 31.6% of new TB patients, and 35.8% of previously treated TB patients had MDR TB. Because national surveillance was conducted on smear-positive samples only, we compared the proportion of MDR TB among patients with smear-positive results in our cohort to prevalence of MDR TB from regional surveillance estimates. As shown in Tables 4 and 5, our cohort showed higher risk for MDR TB among new TB patients and previously treated TB patients.

**Table 3:** Prevalence estimates of TB drug resistance from national and regional surveillance data, and proportion of drug resistance in study cohort, Lima, Peru, 2005–2008*

Cohort	Total no. patients	No. (%) susceptible to INH and RIF	No. (%) monoresistant to INH or RIF	No. (%) MDR TB
Peruvian NTP drug-resistance surveillance, national data (*21*)				
All TB patients	2,167	1,829 (84.4)	158 (7.3)	180 (8.3)
New TB patients	1,816	1,597 (87.9)	124 (6.8)	95 (5.2)
Previously treated patients	351	232 (66.1)	34 (9.7)	85 (24.2)
Peruvian NTP drug-resistance surveillance, regional data corresponding to study area†				
All TB patients	580	467 (80.5)	41 (7.1)	72 (12.4)
New TB patients	476	396 (83.2)	33 (6.9)	47 (9.9)
Previously treated patients	104	71 (68.3)	8 (7.7)	25 (24.0)
Study cohort				
All TB patients	1,241	661 (53.3)	161 (13.0)	419 (33.8)
New TB patients	612	333 (54.4)	84 (13.7)	195 (31.9)
Previously treated patients	629	328 (52.2)	77 (12.2)	224 (35.6)
Study cohort, smear-positive samples only				
All TB patients	1,114	581 (52.2)	143 (12.8)	390 (35.0)
New TB patients	531	278 (52.4)	73 (13.8)	180 (33.9)
Previously treated patients	583	303 (52.0)	70 (12.0)	210 (36.0)

*TB, tuberculosis; INH, isoniazid; RIF, rifampin; MDR, multidrug resistant; NTP, National Tuberculosis Control Program. †Ministerio de Salud, Peru, unpub. data.

**Table 4:** MDR TB among new smear-positive TB patients compared with regional surveillance prevalence estimates, by NTP risk group, Lima, Peru, 2005–2008*

Risk factor	Total no. patients	No. (%) MDR TB	Odds ratio (95% CI)
New smear-positive TB patients in NTP regional surveillance data	476	47 (9.9)	
New smear-positive TB patients in study cohort	531	180 (33.9)	4.68 (3.30–6.65)
HIV positive	46	8 (17.4)	1.92 (0.85– 4.36)
Diabetes mellitus	107	18 (16.8)	1.85 (1.02–3.33)
Chronic corticosteroid therapy	4	0	NA
Other immunosuppression	5	0	NA
Adverse reaction	4	1 (25.0)	3.04 (0.06–38.63)
Previous hospitalization within the past 2 y with duration >15 d	5	2 (40.0)	6.09 (0.49–54.15)
Health care worker during the past 2 y	24	4 (16.7)	1.83 (0.60–5.57)
Health sciences student during the past 2 y	29	5 (17.2)	1.90 (0.69–5.22)
Prisoner during the past 2 y	27	4 (14.8)	1.59 (0.53–4.79)
Adult patient with household contact risk factor(s)†	170	57 (33.5)	4.60 (2.97–7.14)
Pediatric patient with household contact risk factor(s)†	13	7 (53.9)	10.65 (2.90–39.71)
Private or self-administered treatment	2	2 (100.0)	NA
Sputum positive during second or third month of category I treatment	105	70 (66.7)	18.26 (11.01–30.26)
Sputum positive during second or third month of category II treatment	1	1 (100.0)	NA

*MDR, multidrug resistant; TB, tuberculosis; NTP, National Tuberculosis Control Program; CI, confidence interval; NA, not applicable. †Household contact risk factors are defined as household contact with a patient with known MDR TB, with a patient who showed TB treatment failure in the past 2 y, or with a patient being treated with second-line TB drugs.

**Table 5:** MDR TB among previously treated smear-positive TB patients compared with regional surveillance prevalence estimates, by NTP risk group, Lima, Peru, 2005–2008*

Risk factor	Total no. patients	No. (%) MDR TB	Odds ratio (95% CI)
Previously treated smear-positive TB patients in NTP regional surveillance data	104	25 (24.0)	
Previously treated smear-positive TB patients in study cohort	583	210 (36.0)	1.78 (1.10–2.88)
HIV positive	36	12 (33.3)	1.58 (0.69**–**3.61)
Diabetes mellitus	30	10 (33.3)	1.58 (0.65**–**3.82)
Adverse reaction	13	1 (7.7)	0.26 (0.01–1.97)
Previous hospitalization within the past 2 y with duration >15 d	4	1 (25.0)	1.05 (0.02–13.80)
Health care worker during the past 2 y	4	1 (25.0)	1.05 (0.02–13.80)
Health sciences student during the past 2 y	4	3 (75.0)	9.48 (0.71–503.7)
Prisoner during the past 2 y	24	4 (16.7)	0.63 (0.20–2.02)
Adult case with household contact risk factor(s)†	109	56 (51.4)	3.34 (1.86–6.00)
Pediatric case with household contact risk factor(s)†	7	3 (42.9)	2.37 (0.32–14.92)
Private or self-administered treatment	87	28 (32.2)	1.50 (0.79–2.83)
Sputum positive during second or third month of category I treatment	5	3 (60.0)	4.74 (0.50–58.69)
Sputum positive during second or third month of category II treatment	13	11 (84.6)	17.38 (3.36–166.8)
Failure of category I treatment‡	30	22 (73.3)	8.69 (3.44–21.93)
Relapsed within 6 mo after category I treatment§	65	26 (40.0)	2.11 (1.08–4.12)
Defaulted while receiving category I treatment¶	98	15 (15.3)	0.57 (0.28–1.16)
Failure of category II treatment‡	18	11 (61.1)	4.97 (1.74–14.18)
Relapsed within 6 mo after category II treatment§	8	5 (62.5)	5.27 (0.93–35.66)
Defaulted while receiving category II treatment¶	63	16 (25.4)	1.08 (0.52–2.22)
Chronic treatment (>2 prior treatments)#	253	97 (38.3)	1.96 (1.17–3.29)

*MDR, multidrug resistant; TB, tuberculosis; CI, confidence interval; NTP, National Tuberculosis Control Program. †Household contact risk factors are defined as household contact with a patient with known MDR TB, with a patient who showed TB treatment failure in the past 2 y, or with a patient being treated with second-line TB drugs. ‡Defined as positive smear and/or culture after >4 mo of treatment, or positive smear and/or culture upon finishing treatment. §Defined as recurrence of disease <6 mo after being classified as cured by NTP norms. ¶Defined as not receiving treatment >1 mo upon enrollment into the study. #Defined as a history of >2 previous TB treatments.

When stratifying our cohort by risk group, we found that diabetes mellitus (16.8%), adult (33.5%) or child (53.9%) patients with household contacts with known or suspected MDR TB, and suspected category I failure, i.e., positive smear or culture during the second or third month of category I therapy (66.7%), were associated with significantly higher relative risks of MDR TB among patients with new smear-positive TB, when compared with regional surveillance prevalence estimates. Among the 18 patients with diabetes and new smear-positive MDR TB, 10 (55.6%) had 2 risk factors for MDR TB at the time of enrollment. Of these, 5 (27.8%) had suspected category I failure, 4 (22.2%) were adults with a household contact, and 1 (5.6%) had confirmed category I failure. Breslow-Day tests for homogeneity indicated that the effect of adult household contact on the odds of MDR TB is modified by diabetes (p<0.0001), and that the effect of suspected category I failure on the odds of MDR TB is modified by diabetes (p = 0.0113). One patient with new smear-positive TB was suspected of failing category II treatment (i.e., positive smear or culture during the second or third month of category II therapy); this same patient met the risk group criteria for adult household contact and private or self-administered treatment. Among previously treated patients with smear-positive TB, the following factors were significantly associated with a higher relative risk for MDR TB, compared with regional surveillance prevalence estimates: adult household contact (51.4%), failure of category I treatment (73.3%), early relapse after category I treatment (40.0%), suspected (84.6%) or confirmed (61.1%) failure of category II treatment, and history of >2 previous TB treatments (38.3%).

## Discussion

We describe the proportion of drug resistance among TB patients as detected by using the screening strategy for MDR TB instituted in Lima, Peru, starting in 2005. When these data were compared with nearly concurrent population-based surveillance data, the proportion of MDR TB among new and previously treated TB cases was found to be significantly higher, indicating that screening high-risk patients may be an effective strategy. The proportion of MDR TB detected among patients with new smear-positive TB is comparable to that among previously treated patients with smear-positive TB in the cohort (p = 0.458). In the Peruvian NTP surveillance regional data corresponding to the study area, the prevalence of MDR TB among patients with new smear-positive TB was significantly lower than the prevalence among previously treated patients with smear-positive TB (p<0.001). This finding shows that the strategy implemented in Lima was especially effective in detecting MDR TB among patients with new smear-positive TB.

The risk groups with the highest rates of MDR TB were those with diabetes mellitus, adults or children with household contacts with known or suspected TB, suspected failure of category I or II treatment (i.e., positive smear or culture during the second or third month of therapy), failure or early relapse to category I treatment, failure of category II treatment, and multiple (>2) previous TB treatments. Ample literature supports these findings in a variety of settings (*22**–**25*). Screening for drug resistance among these groups is easily implemented and should be strongly considered by national TB programs.

In addition to identifying risk groups with high prevalence of MDR TB, other considerations are pertinent to the design of an optimal programmatic strategy. For example, risk groups with a relatively low prevalence of MDR TB may still merit DST if delays in initiation of MDR TB treatment would have severe consequences (e.g., children or HIV-positive patients) or if the absolute number of MDR TB cases within that risk group is substantial (e.g., patients with diabetes). The relative complexity of implementing certain testing strategies is also a consideration. Compared with alternative testing strategies such as universal testing or testing by geographic region, a strategy that focuses on high-risk patients requires training health care workers to screen each TB patient for numerous risk factors. Therefore, case finding may be variable under routine program conditions.

The findings of this evaluation are subject to several limitations. Although our study personnel would visit local health establishments in a purely observational capacity, the frequent visits by data collectors in the health centers could have sensitized health care workers to follow screening and referral protocols more closely than they would have otherwise. In addition, given the use of phenotypic methods, the drug-resistance status of particular isolates could be determined only for culture-positive samples. Although the yield of positive cultures was high for all methods used (67.4% for indirect conventional DST, 78.5% for direct NRA, and 35.3% for largely paucibacillary or smear-negative samples submitted for BACTEC; data not shown), the contribution to relative risk of MDR TB among those without DST results could not be determined. These results call attention to one of the shortcomings of all phenotypic methods, i.e., a substantial fraction of patients never have positive cultures or DST results to confirm the diagnosis or guide therapy, despite being at high risk for having MDR TB. Nonetheless, the yield of positive cultures in our sample is similar to that obtained by programs that have used the same sputum-processing methods (n-acetyl-l-cysteine and centrifugation, with cultivation on LJ medium). Finally, this evaluation was observational in nature and lacks a concurrent comparison group, such as one that had undergone an alternative screening strategy. On the other hand, this study has key strengths. The programmatic nature of this intervention, an active field presence to capture accurate and complete data on a large cohort, and the fortuitous concurrent surveillance study have allowed us to assess the effects of these programmatic efforts to identify patients with MDR TB.

To date, little research has been conducted on the comparative effectiveness of varied approaches to MDR TB screening and treatment referral. To our knowledge, the only study to show clinical results for performing rapid DST is a retrospective study carried out in California, which showed that using a molecular beacon assay led to earlier diagnosis and treatment initiation for MDR TB (*26*). An important aspect of program evaluation is feedback of findings to further improve treatment programs. In Peru, the results of this evaluation have been conveyed to the NTP and NRL. The aim of this communication is to describe an intensive evaluation of one of Peru's public health strategies for improving MDR TB control. In other low- to middle-income countries, similar program evaluations should be implemented to clarify national and regional MDR TB epidemiology, identify key risk groups for MDR TB, and inform national strategies to diagnose and treat MDR TB. Ultimately, the effects of these changes on turn-around time, time to culture conversion, cure rates, and costs will determine the comparative success of these strategies.
